# Life-history traits of *Drosophila melanogaster* populations exhibiting *early* and *late* eclosion chronotypes

**DOI:** 10.1186/s12862-016-0622-3

**Published:** 2016-02-27

**Authors:** K. L. Nikhil, Karatgi Ratna, Vijay Kumar Sharma

**Affiliations:** Chronobiology Laboratory, Evolutionary and Organismal Biology Unit, Jawaharlal Nehru Centre for Advanced Scientific Research, Jakkur, PO Box. 6436, Bangalore, Karnataka 560064 India

**Keywords:** Circadian, Adaptive significance, Fitness, Laboratory selection, Life-history evolution

## Abstract

**Background:**

The hypothesis that circadian clocks confer adaptive advantage to organisms has been proposed based on its ubiquity across almost all levels of complexity and organization of life-forms. This thought has received considerable attention, and studies employing diverse strategies have attempted to investigate it. However, only a handful of them have examined how selection for circadian clock controlled rhythmic behaviors influences life-history traits which are known to influence Darwinian fitness. The ‘early’ and ‘late’ chronotypes are amongst the most widely studied circadian phenotypes; however, life-history traits associated with these chronotypes, and their consequences on Darwinian fitness remain largely unexplored, primarily due to the lack of a suitable model system. Here we studied several life-history traits of *Drosophila melanogaster* populations that were subjected to laboratory selection for morning (*early*) and evening (*late*) emergence.

**Results:**

We report that the *late* eclosion chronotypes evolved longer pre-adult duration as compared to the *early* eclosion chronotypes both under light/dark (LD) and constant dark (DD) conditions, and these differences appear to be mediated by both clock dependent and independent mechanisms. Furthermore, longer pre-adult duration in the *late* chronotypes does not lead to higher body-mass at pupariation or eclosion, but the *late* females were significantly more fecund and lived significantly shorter as compared to the *early* females.

**Conclusions:**

Coevolution of multiple life-history traits in response to selection on timing of eclosion highlights correlations of the genetic architecture governing timing of eclosion with that of fitness components which suggests that timing ecologically relevant behaviors at specific time of the day might confer adaptive advantage.

**Electronic supplementary material:**

The online version of this article (doi:10.1186/s12862-016-0622-3) contains supplementary material, which is available to authorized users.

## Background

It is believed that circadian timekeeping mechanisms underlying rhythmic processes provide adaptive advantage to organisms [1–7], and this has prompted studies employing a variety of strategies to examine the adaptive benefits of possessing functional circadian clocks. Surgical ablation of the mammalian ‘master circadian clock’ - suprachiasmatic nucleus [8], and genetic manipulation of circadian clocks in fruit flies *Drosophila melanogaster* [9] which are known to drive loss of rhythmicity in several key circadian behaviors result in reduced survivorship [10–13]. Environmentally induced or naturally occurring circadian dysfunction has also been reported to reduce longevity in *D. melanogaster* [14, 15]. Beaver et al. [16, 17] reported that *D. melanogaster* strains carrying loss-of-function mutation in two core clock genes exhibit reduced reproductive output. In addition, studies on organisms inhabiting different latitudes, as well as those living in constant conditions reported large variation in circadian phenotypes in accordance to their local habitats, suggesting that the underlying clocks may have evolved as an adaptation to the presence or absence of cyclic environmental conditions [6, 18–28]. Nevertheless, conclusions drawn from such studies are limited by the lack of adequate information about the ancestry, population size and history of the environmental conditions pertaining to the organism’s ecology [6].

The eclosion waveform of *D. melanogaster* comprises a primary peak at dawn (under natural conditions) or around night-day transition (under laboratory light/dark cycles) which gradually reduces through the day with little or no eclosion occurring at night (Additional file 1: Figure S1; [9, 29]). This restriction/gating of eclosion primarily around dawn is hypothesized to be an adaptation to avoid desiccation of pharate adults by high temperature and low humidity prevailing during the rest of the day [3], partly supported by the results of a recent study [30]. Laboratory selection approach has been previously adopted to study how circadian clocks evolve in response to selection for time/phase of eclosion. Selection for ‘early’ and ‘late’ emerging strains of *Drosophila pseudoobscura* and moth *Pectinophora gossypiella* under LD12:12 (12 h of light and dark cycles each) resulted in the evolution of divergent phase of eclosion (4 h in *D. pseudoobscura* and 5 h in *P. gossypiella*) [31, 32]. As a correlated response, the early flies in both studies evolved longer circadian clock period while the late flies evolved shorter period. However, these studies suffered from some major shortcomings such as lack of population level replication, details of population ancestry and selection protocols employed (population maintenance methodology, population size and sex ratio) which are known to considerably modify the evolutionary trajectories in response to selection; and thus might have led to misinterpretation of the observed responses to selection (reviewed in [6]). Although these studies suggest that circadian clocks might have evolved to ensure temporal order in behavior and physiology thus enhancing Darwinian fitness (reviewed in [6]), our understanding of how selection for timing of clock controlled behaviors influence life-history traits remains nominal.

To explore the evolutionary trajectory of circadian clocks in response to selection for timing of eclosion, we initiated a long-term study on *D. melanogaster* populations by imposing selection for eclosion during early morning and late evening hours, which is in contrast to the usual time of eclosion in this species. From a set of 4 ancestral *control* populations we derived 8 populations - 4 replicate *early* populations using flies that eclose early in the morning and 4 replicate *late* populations using flies that eclose late in the evening (Additional file 1: Figure S2; see materials and methods for detailed selection protocol). Consequently, the *early*_*1-4*_ and the *late*_*1-4*_ populations evolved significantly higher morning and evening eclosion respectively relative to the *control*_*1-4*_ populations, and exhibited several properties analogous to the well-known ‘morning/early’ and ‘evening/late’ chronotypes in humans. Similar to the ‘early’ and the ‘late’ human chronotypes [33–35], the *early* and the *late Drosophila* populations evolved shorter and longer clock periods respectively with the *control* populations exhibiting intermediate period [36], and also exhibited diverged photic phase response curves (PRCs) for both eclosion [36] and activity/rest rhythms [37]. These results indicate that circadian clocks of the two sets of populations ‘entrain’ differently to LD cycles, or in other words, they are differentially sensitive/interact differentially with LD cycles. This is corroborated by the results of a previous study which reported that the *early* populations are sensitive to light primarily in the evening while the *late* populations are sensitive to light primarily in the morning [38]. Collectively, these studies suggest that divergent coevolution of circadian clocks in the *early* and the *late* populations might mediate differential interaction/entrainment to regulate time of eclosion.

In the present study, we used the *early* and the *late* populations to examine genetic correlations between mechanisms that underlie eclosion at a specific time of the day and that of various pre-adult (egg-to-puparium and egg-to-adult duration, egg-to-puparium and egg-to-adult survivorship, and puparial dry-weight) as well as adult life-history traits (dry-weight at eclosion, fecundity, pre- and post-fecundity assay dry-weight, and longevity). As discussed earlier, the *early* and the *late* eclosion chronotypes have been shown to be associated with different circadian clock period and differential entrainment to LD cycles, and pre-adult traits such as egg-to-adult duration is known to be correlated with circadian clock period. Therefore, to assess the relative contribution of circadian clock period and differential entrainment to LD cycles in driving life-history trait differences between the *early* and the *late* populations, we performed some of our experiments under both LD12:12 as well as constant darkness (DD). The rationale being that if differences in life-history traits between the *early* and the *late* populations are solely determined by circadian clock period as can be observed under DD when the clock is not under the influence of LD cycles, such differences would either decrease or cease to exist because clock period of all the populations would be held at 24 h in LD 12:12 by virtue of entrainment [8]. Persistence of differences between populations under both light regimes would imply that the observed life-history trait differences are also driven by clock independent mechanisms.

As mentioned earlier, since *D. melanogaster* eclose predominantly during ‘dawn’, eclosion at other times of the day is considered to be maladaptive (Additional file 1: Figure S1; [3]). If this is true, then the proportion of individuals which normally eclose early in the morning in the c*ontrol* populations might also differ in terms of fitness from those that eclose late in the evening. To test for such a possibility, one generation before the assays we derived 8 additional populations from the *controls* ─ 4 populations comprising individuals emerging early in the morning, referred to as the *early-control*, and similarly, 4 populations comprising individuals emerging late in the evening, referred to as the *late*-*control*. Also, the *early-control* and the *late-control* populations are likely to reveal whether the observed differences in fitness measures between the *early* and the *late* populations (if any) are indeed evolved responses to the selection imposed on the timing of eclosion, or are merely environment-driven.

We report that the *late* populations have evolved significantly longer median egg-to-puparium duration leading to longer egg-to-adult duration, are more fecund around day 11 post-emergence which is the usual day for egg collection as per the selection protocol (see materials and methods), and also exhibit reduced median longevity as compared to the *early* populations, whereas the *early-control* and the *late-control* populations did not differ in the aforesaid life-history traits thus suggesting that the observed differences between the selected populations (*early* and *late*) are evolutionary responses to selection for timing of eclosion. Also, even though the *early* populations differed significantly from the *late* populations, they were similar to the *control* populations for most of the traits assayed, the possible reasons for which are discussed later.

## Results

### Egg-to-puparium duration

**Fig. 1:**
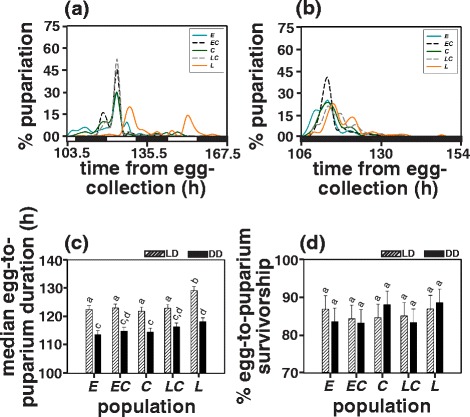
Percentage pupariation as a function of time (in hours) from egg-collection in **a** LD12:12 and **b** DD for the *early* (*E)*, the *early-control* (*EC*), the *control* (*C*), the *late-control* (*LC*) and the *late* (*L*) populations. The black and white bars at the bottom represent night and day respectively. **c** Median egg-to-puparium duration (time taken for 50 % of total pupariation events) of all populations in LD12:12 and DD, and **d** Percentage egg-to-puparium survivorship values in LD12:12 and DD. Error bars for panels c and d indicate 95 % confidence intervals calculated by method of Tukey’s HSD. Bars sharing same letters do not differ statistically while those with different letters are significantly different from each other

ANOVA on median egg-to-puparium duration showed statistically significant effect of population, light regime and population × light regime interaction (Table 1a). Across light regime comparisons revealed that egg-to-puparium duration for all the populations was significantly longer (by 8.4 h or 7 %) in LD12:12 as compared to that in DD thus highlighting the delaying effect of LD cycles on egg-to-puparium duration (Fig. 1a-c; Additional file 1: Figure S3; Additional file 1: Table S1).

**Table 1:** Summary of results of ANOVA on (a) median egg-to-puparium duration, (b) arc-sine square root transformed egg-to-puparium survivorship, (c) median egg-to-adult duration, (d) arc-sine square root transformed egg-to-adult survivorship, (e) dry-weight at pupariation, and (f) dry-weight at eclosion values of all populations under LD12:12 and DD light regimes. Summary of results of ANOVA on (g) average eggs laid/female, (h) dry-weight at pre- and post fecundity assay stages, (i) log transformed fecundity per unit dry-weight loss, and (j) median longevity of virgin males and females of all populations under LD12:12

Trait	Effect	*df*	MS	*F*	*p*
(a) median egg-topuparium duration	population	4	42.60	48.80	<0.0001
	light regime	1	704.4	2261.7	<0.0001
	population × light regime	4	5.70	3.70	0.0346
(b) egg-to-puparium survivorship	population	4	14.70	1.47	0.2711
	light regime	1	0.03	0.004	0.9540
	population × light regime	4	10.6	1.05	0.4218
(c) median egg-to-adult duration	population	4	58.00	20.00	<0.0001
	light regime	1	2588	2189	<0.0002
	population × light regime	4	8.00	4.00	0.0207
(d) egg-to-adult survivorship	population	4	3.20	0.38	0.8179
	light regime	1	22.7	40.52	0.0078
	population × light regime	4	8.30	3.78	0.0326
(e) dry-weight at pupariation	population	4	142	4.7	0.0167
	light regime	1	13231	136.3	0.0013
	population × light regime	4	40	00.60	0.6407
(f) dry-weight at eclosion	population	4	71	2.4	0.1107
	light regime	1	2505	150.9	0.0011
	population × light regime	4	34	2.0	0.1612
(g) eggs laid/female	population	4	9.31	24.315	<0.0001
(h) pre- and post-fecundity dry-weight	population	4	53	2.00	0.1567
	stage	1	27465	6835.6	<0.0001
	population × stage	4	249	18.10	<0.0001
(i) fecundity/unit dry-weight loss	population	4	0.002	0.43	<0.7782
(j) longevity	population	4	17.99	17.22	<0.0001
	sex	1	83.45	70.88	0.0035
	population × sex	4	11.44	5.27	0.0011

In LD12:12, the *late* populations had a significantly longer (by 6.5 h or 5.4 %) egg-to-puparium duration (129.11 h) as compared to all other populations (*early* = 122.43 h, *early-control* = 123.01 h, *control* = 121.84 h and *late-control* = 122.89 h) while that for the remaining four sets of populations did not differ among each other (Fig. 1a, c; Additional file 1: Figure S3; Additional file 1: Table S1).

In DD, the *late* populations took significantly longer (by 5 h or 3.6 %) to pupariate (118.14 h) as compared to the *early* (113.57 h) and the *control* (114.47 h) populations but did not differ from the *early-control* (114.83 h) and the *late-control* (116.32 h) populations, whereas none of the other sets of populations differed among each other (Fig. 1b, c; Additional file 1: Figure S3; Additional file 1: Table S1).

### Egg-to-puparium survivorship

ANOVA on egg-to-puparium survivorship revealed that the effect of population, light regime and population × light regime interaction was statistically not significant (Table 1b), indicating that the populations did not differ in their egg-to-puparium survivorship both within and across light regimes.

The average egg-to-puparium survivorship across populations was 85.56 ± 1.24 % (mean ± SD) in LD12:12 (*early* = 86.86 %, *early-control* = 84.33 %, *control* = 84.60 %, *late-control* = 85.08 % and *late* = 86.93 %) and 85.35 ± 1.72 % (mean ± SD) in DD (*early* = 83.56 %, *early-control* = 83.18 %, *control* = 88.03 %, *late-control* = 83.36 % and *late* = 88.61 %; Fig. 1d; Additional file 1: Table S2).

### Egg-to-adult duration

**Fig. 2:**
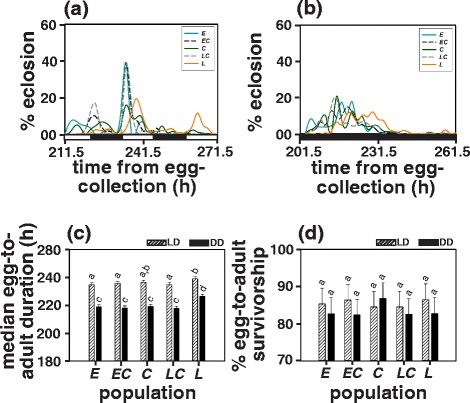
Percentage of flies eclosing as a function of time (in hours) from egg-collection in **a** LD12:12 and **b** DD for the *early* (*E)*, the *early-control* (*EC*), the *control* (*C*), the *late-control* (*LC*) and the *late* (*L*) populations. The black and white bars at the bottom represent night and day respectively. **c** Median egg-to-adult duration of all populations in LD12:12 and DD, and **d** Percentage egg-to-adult survivorship values of all populations in LD12:12 and DD. All other details are same as in Fig. 1

ANOVA on median egg-to-adult duration revealed statistically significant effect of population, light regime and population × light regime interaction (Table 1c). As observed for egg-to-puparium duration, the egg-to-adult duration in LD12:12 was also significantly longer (by 16 h or 7.5 %) for all the populations as compared to that in DD (Fig. 2a-c; Additional file 1: Figure S4).

In LD12:12, egg-to-adult duration of the *late* population (239.15 h) was significantly longer (by 4 h or 1.9 %) than all populations (*early* = 234.69 h, *early-control* = 235.70 h and *late-control* = 234.68 h) except the *control* (236.44 h) populations whereas that of all other populations (*early, early-control*, *control* and *late-control*) did not differ significantly among each other (Fig. 2a, c; Additional file 1: Figure S4; Additional file 1: Table S1).

Under DD, the *late* populations exhibited a significantly longer (by 8 h or 3.5 %) egg-to-adult duration (226.41 h) as compared to all other populations (*early* = 218.90 h, *early-control* = 217.96 h and *late-control* = 217.79 h) except the *controls* (219.18 h; Fig. 2b, c; Additional file 1: Figure S4; Additional file 1: Table S1).

### Egg-to-adult survivorship

ANOVA on egg-to-adult survivorship revealed statistically significant effect of light regime and population × light regime interaction but not of population (Table 1d). However, post hoc multiple comparisons using Tukey’s HSD did not reveal any statistically significant difference in egg-to-adult survivorship either across LD12:12 (*early* = 85.33 %, *early-control* = 86.38 %, *control* = 84.51 %, *late-control* = 84.50 % and *late* = 86.48 %) and DD (*early* = 82.74 %, *early-control* = 82.37 %, control = 86.82 %, *late-control* = 82.52 % and *late* = 82.76 %) light regimes or across populations within a light regime (Fig. 2d; Additional file 1: Table S2).

### Dry-weight

Since the *late* populations exhibited significantly longer egg-to-puparium and egg-to-adult duration, we further tested if this lengthening of pre-adult developmental duration translated to higher dry-weight at pupariation and eclosion.

**Fig. 3:**
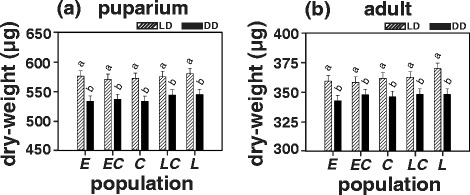
Dry-weight per individual at **a** pupariation, and **b** eclosion for the *early* (*E)*, the *early-control* (*EC*), the *control* (*C*), the *late-control* (*LC*) and the *late* (*L*) populations in LD12:12 and DD light regimes. All other details are same as in Fig. 1

ANOVA on pupal dry-weight revealed statistically significant effect of population and light regime but not of population × light regime interaction (Table 1e). In accordance with the difference in egg-to-puparium duration between light regimes, the pupal dry-weight was found to be significantly higher (on an average by 6.3 %) in LD12:12 (*early* = 576.16 μg, *early-control* = 570.53 μg, *control* = 572.17 μg, *late-control =* 575.11 μg and *late* = 580.16 μg; Fig. 3a; Additional file 1: Table S3) as compared to that in DD (*early* = 533.52 μg, *early-control* = 536.46 μg, *control* = 533.33 μg, *late-control =* 544.07 μg and *late* = 544.83 μg; Fig. 3a; Additional file 1: Table S3) whereas no difference was observed between populations within either of the light regimes.

ANOVA on dry-weight at eclosion reported statistically significant effect of light regime but not of population or population × light regime interaction (Table 1f). In accordance with egg-to-adult duration differences across light regimes, dry-weight at eclosion was found to be significantly higher (on an average by 4.35 %) in LD12:12 (*early* = 359.39 μg, *early-control* = 358.30 μg, *control* = 361.71 μg, *late-control =* 362.64 μg and *late* = 369.94 μg; Fig. 3b; Additional file 1: Table S3) as compared to that in DD (*early* = 342.63 μg*, early-control* = 347.85 μg, *control* = 346.19 μg, *late-control =* 348.12 μg and *late* = 348.06 μg; Fig. 3b; Additional file 1: Table S3) whereas the populations did not differ among each other in either of the light regimes.

### Fecundity

**Fig. 4:**
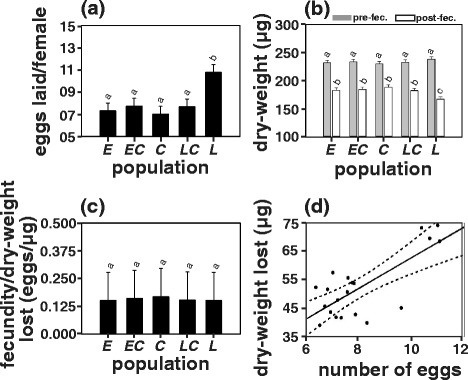
**a** Average fecundity per female on day 11 (post-eclosion), **b** dry-weight per female at pre- and post-fecundity stages, and **c** fecundity per unit dry-weight loss (difference in pre- and post-fecundity dry-weight) for the *early* (*E)*, the *early-control* (*EC*), the *control* (*C*), the *late-control* (*LC*) and the *late* (*L*) populations in LD12:12. Error bars indicate 95 % confidence intervals calculated by method of Tukey’s HSD. **d** Pearson correlation of average fecundity/female with that of dry-weight lost during the fecundity assay (*r* = +0.75, *p* < 0.0001). The data points for correlation were obtained by pooling values across all 20 populations. The dotted line indicates 95 % confidence interval. Fecundity and dry-weight measurements assays were performed only in LD12:12. Bars sharing same letters do not differ statistically while those with different letters are significantly different from each other

ANOVA on average fecundity data revealed a statistically significant effect of population (Table 1g). Fecundity of the *late* populations (10.80 eggs/fly) was significantly higher (by 32 %) as compared to that of the other populations (*early* = 7.32 eggs/fly, *early-control* = 7.74 eggs/fly, *control* = 7.01 eggs/fly and *late-control* = 7.68 eggs/fly), whereas none of the other populations differed significantly among each other (Fig. 4a; Additional file 1: Table S4).

### Pre- and post-fecundity assay dry-weights

ANOVA on female dry-weight measurements at pre- and post-fecundity assay stages showed statistically significant effect of stage (pre/post-fecundity assay) and population × stage interaction but not of population (Table 1h). Post hoc multiple comparisons revealed that post-fecundity assay dry-weight of all the populations was reduced by about 52.40 μg (22 %; Fig. 4b) as compared to the pre-fecundity assay dry-weight. Pre-fecundity assay dry-weight did not differ statistically between populations (*early* = 231.95 μg*, early-control* = 233.62 μg, *control* = 229.95 μg, *late-control =* 232.87 μg and *late* = 238.45 μg; Table 1h; Additional file 1: Table S4) but post-fecundity assay dry-weight of the *late* populations (167.16 μg) was significantly lower (by 17 μg or ~10 %) as compared to that of all other populations (*early* = 182.79 μg, *early-control* = 184.30 μg, *control* = 188.41 μg and *late-control* =182.16 μg; Fig. 4b; Table 1h; Additional file 1: Table S4).

### Fecundity per unit loss in dry-weight

When normalized by the dry-weight lost (difference in pre- and post-fecundity assay dry-weight), fecundity per unit dry-weight lost did not differ statistically across populations (*early* = 0.15 eggs/μg, *early-control* = 0.16 eggs/μg, *control* = 0.17 eggs/μg, *late-control* = 0.15 eggs/μg and *late* = 0.15 eggs/μg; Fig. 4c; Table 1i), suggesting that although the *late* populations were more fecund they lose more dry-weight due to the higher number of eggs laid. As an additional confirmation of this, we performed a linear correlation between egg output and dry-weight loss by pooling data from all the populations, and found that the two were significantly positively correlated (*r* = +0.75, *p* < 0.0001; Fig. 4d).

### Longevity

**Fig. 5:**
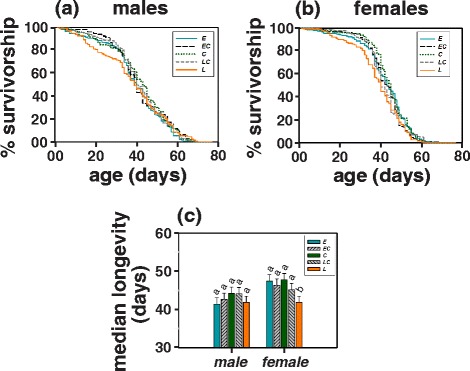
Survivorship curves of **a** virgin males and **b** virgin females of the *early* (*E)*, the *early-control* (*EC*), the *control* (*C*), the *late-control* (*LC*) and the *late* (*L*) populations in LD12:12. **c** Median longevity (time taken for 50 % of individuals to die) of virgin males and females of all populations in LD12:12. All other details are same as in Fig. 1

ANOVA on median longevity reported statistically significant effect of population, sex and population × sex interaction (Table 1j). With the exception of the *late* populations where individuals of both the sexes had an average median longevity of 41.89 ± 0.004 days (mean ± SD), the average female longevity of all the other populations (*early* = 47.5 days, *early-control* = 46.38 days, *control* = 47.81 days and *late-control* = 45.22 days; Fig. 5; Additional file 1: Table S4) was ~7 % higher than that of the males (*early* = 41.41 days, *early-control* = 42.67 days, *control* = 44.25 days and *late-control* = 44.13 days; Fig. 5; Additional file 1: Table S4).

Within sex comparisons revealed that females of the *late* populations exhibited significantly shorter (~12 %) median longevity as compared to females of the other populations with the exception of the *late-control* females which did not differ statistically from the *late* populations. The median male longevity was not observed to differ statistically across populations (Fig. 5; Additional file 1: Table S4).

## Discussion

We observe that the *late* populations evolved longer egg-to-puparium and egg-to-adult duration as compared to the *early* populations thus highlighting an association between eclosion chronotype and pre-adult developmental duration (Figs. 1a-c, 2a-c). Under LD12:12, the difference in median egg-to-puparium duration between the *late* and the *early* populations is ~7 h, which is reduced to ~3 h at eclosion (Figs. 1c, 2c). One possible reason for this might be a genotype dependent effect of light on pupal development as also suggested by the median egg-to-puarium duration of the *late* populations which differs from all other populations under LD12:12 but not DD (Fig. 1c). Alternatively, under LD cycles, timing of eclosion is known to be governed by a circadian clock component as well as a clock independent masking response to lights-ON as discussed with respect to the same populations in a previous study [39]. Thus, in addition to the clock determined time of eclosion, masking response to light, which results in an additional burst of eclosion immediately following lights-ON might have reduced the median pre-adult duration. This is further supported by our observation under DD that ~4 h difference in egg-to-puparium duration between the *late* and the *early* populations increases to ~7 h at eclosion (Figs. 1c, 2c). Thus, the observed reduction in median egg-to-adult duration under LD12:12 may be a result of the combination of both (a) artefact of masking response to lights-ON which is clearly absent under DD and (b) differential effect of light on pupal development, which remains to be addressed further. In addition to the divergent eclosion chronotypes, the *early* and the *late* populations have also evolved shorter and longer clock periods differing by 40 min [36, 39] which suggests a correlation between emergence chronotype and circadian clock period. Such correlations have been reported earlier in the melon fly, *Bactrocera cucurbitae* [40, 41], and between clock period and egg-to-adult duration in fruit flies *D. melanogaster* [42–44], suggesting that clock period differences influence pre-adult developmental rates. In DD, egg-to-puparium duration of the *late* populations was 118.14 h (4.9 days) and egg-to-adult duration was 226.41 h (9.4 days) as opposed to 113.56 h (4.7 days) and 218.90 h (9.1 days) respectively of the *early* populations. If the pre-adult duration of the *early* and the *late* populations was entirely driven by circadian clock period difference, under DD the *early* and the *late* populations would drift apart by 0.66 h (40 min) every day, and consequently the two populations would exhibit a 3.12 h difference in egg-to-puparium duration (in 4.7 days which is equal to the time taken by the *early* population to pupariate) and 6.01 h difference in egg-to-adult duration (in 9.12 days) which is considerably smaller than that observed empirically (Figs. 1a, c; 2a, c). Since eggs for the egg-to-puparium and egg-to-adult duration assays were collected from all populations at the same time (thus were age matched), the observed differences in pre-adult duration between the *early* and the *late* populations are unlikely to be due to the differences in the age of eggs. Moreover, the time of egg-collection or the age of eggs does not alter the difference in egg-to-adult duration between the *early* and the *late* populations [45]. Taken together these results suggest that difference in pre-adult developmental rates of the *early* and the *late* populations is not entirely circadian clock driven, and may also involve clock independent mechanisms which might drive differential interaction with LD cycles (significant population × light regime interaction reported in Table 1a, c).

Furthermore, light mediated enhancement in the pre-adult developmental rates is apparent as both egg-to-puparium and egg-to-adult duration of all the populations was 7-7.5 % longer in LD12:12 as compared to DD (Figs. 1a-c, 2a-c; Additional file 1: Figure S3, Additional file 1: Figure S4; Additional file 1: Table S1). While effect of light on pre-adult duration has been documented earlier [46, 47], precise mechanisms underlying such effects remains to be explored. The timing of eclosion in *Drosophila* is believed to depend upon a number of factors including the developmental state of the fly, the phase and period of circadian rhythm, hormonal cascade, and environmental condition [48, 49]. The LD cycles interact with the circadian clock controlled gate of eclosion such that even if flies have completed development, they are allowed to eclose only during certain time of the day and not merely in accordance with their developmental state, and consequently the time of eclosion is delayed in LD12:12 as compared to DD [47–50]. Additionally, the time of eclosion on a given day is also a function of the circadian clock period such that individuals with shorter period eclose earlier than those with longer clock period [51]. This further supports the notion that pre-adult development in *D. melanogaster* is probably mediated by the interaction of circadian clocks with LD cycles.

Differences in pre-adult developmental rates of the *early* and the *late* populations do not seem to influence their egg-to-puparium and egg-to-adult survivorship; nor do the light regime mediated differences affect pre-adult survivorship (Figs. 1d, 2d; Additional file 1: Table S2). This might be due to the magnitude of difference in egg-to-puparium or egg-to-adult duration between the populations not being large enough to influence egg-to-puparium and egg-to-adult survivorship.

Although the *late* populations have evolved longer pre-adult developmental duration, their body-weight at pupariation and at eclosion did not differ from that of all the other populations (Fig. 3; Additional file 1: Table S3). However, dry-weight of puparia and adults were found to be significantly higher for all the populations in LD12:12 as compared to DD (Fig. 3b; Additional file 1: Table S3) which is not surprising as egg-to-puparium and egg-to-adult duration is significantly enhanced in LD12:12 as compared to DD (Figs. 1, 2).

Coevolution of pre-adult life-history traits in response to selection for timing of eclosion is intuitive, as changes in pre-adult stages can directly affect the time course and waveform of eclosion. It would be interesting to know whether selection for eclosion at different time of the day also led to correlated changes in adult life-history traits that may not necessarily influence eclosion time but would highlight the underlying genetic correlation. In this regard, we observed that the *late* populations exhibited a significantly higher fecundity as compared to all other populations (Fig. 4a; Additional file 1: Table S4). In *D. melanogaster*, pre-adult developmental duration is known to be correlated with fecundity, and delayed development is associated with higher dry-weight, and in turn with higher fecundity [52–54]. This does not appear to be the case in the *late* populations since neither their dry-weight at eclosion nor pre-fecundity assay dry-weight differed from the other populations (Fig. 4b; Additional file 1: Table S4). However, post-fecundity assay dry-weight of the *late* populations was significantly lower as compared to that of the other populations (Fig. 4b; Additional file 1: Table S4), but when normalized by the loss of dry-weight (difference in pre- and post-fecundity assay dry-weight), fecundity per unit dry-weight lost was similar for all populations (Fig. 4c). Therefore, a significant reduction in post-fecundity assay dry-weight in the *late* populations appears to be a consequence of higher number of eggs laid as also substantiated by a significant correlation observed between the number of eggs laid and dry-weight lost (Fig. 4d). Therefore, contrary to the well-known positive correlation between pre-adult developmental duration, dry-weight and fecundity, our results suggest that the observed higher fecundity in the *late* populations is not due to higher dry-weight attained because of the delay in the timing of eclosion but may be due to other mechanisms such as pleiotropy or mutation accumulation [55]. Alternatively, higher fecundity in the *late* populations might have evolved as an artefact of the nature of selection protocol employed. For instance, to ensure that the number of adults in all populations is ~1200, every generation we collect larger number of eggs for the *late* populations followed by relatively smaller number of eggs for the *early* populations as compared to the *control* populations (see materials and methods) which is 24 vials per replicate population for the *early* populations, 48 vials for the *late* populations as opposed to 16 vials for the *control* populations with each vial housing approximately 300 eggs. Therefore, the number of eggs collected from the *late* populations (~14400 eggs) is approximately twice that of the *early* (~7200 eggs) and thrice that of the *control* (~4800 eggs) populations. This might have led to an inadvertent selection for higher fecundity in the *late* populations, and also, possibly as a consequence of higher effective population size (*N*_e_) the *late* populations might experience relatively lower extent of inbreeding depression followed by the *early* populations, with the *control* populations experiencing highest degree of inbreeding depression. If this were to be true, then the *early* populations would also be expected to exhibit higher fecundity as compared to the *control* populations*,* but that does not seem to be the case*.* Therefore, it is unlikely that this reasoning can account for the evolution of higher fecundity in the *late* populations even though it cannot be entirely disregarded. However, the possibility of such a scenario can also be clarified by a cross experiment between the *early* and the *late* populations. Additionally, given that fecundity in *Drosophila* is not constant across lifespan, the difference in fecundity between the populations observed on days 10-12 post-eclosion might also vary across different ages. For instance, in light of the results from a previous study [55]*,* it is also possible that the *early* populations which exhibit significantly lower fecundity on days 10-12 might otherwise exhibit higher fecundity at an earlier stage and *vice versa* for the *late* populations. However, since the fly populations used in the current study are maintained on a non-overlapping 21 day generation cycle (see materials and methods) wherein eggs laid only on the day 11 of adulthood are used for the next generation, only eggs laid around this day determines the populations’ fitness in this regime. Therefore, fecundity during other life-stages is irrelevant under the currently discussed regimen but nevertheless will be interesting to examine.

Further, we found that females of the *late* populations live significantly shorter as compared to those from the *early* and the *control* populations, while no difference in longevity was observed between males (Fig. 5a-c). The reduction in longevity of the *late* females as compared to the *early* and the *control* females was consistently observed in four replicate populations maintained under similar environmental conditions. In light of fecundity and dry-weight results, the observed reduction in longevity of the *late* females appears to represent the classic trade-off between fecundity and adult lifespan due to the antagonistic pleiotropic effects of the underlying genes [56–58]. However, since the results presented here are on virgins, the observed reduction in longevity cannot be explained entirely by such a trade-off. Therefore, even though higher reproductive output may have evolved as an artefact of the selection protocol, reduced longevity in the *late* females as compared to the *early* and the *control* females may have evolved as a correlated response to selection for late evening eclosion and not directly as a consequence of higher fecundity. Interestingly, we also observe that the reduced longevity in the females of the *late* populations is primarily a consequence of early death (around days 20-40) and not during the later life-stages, and a similar trend is also observed in the males. However, the possible reason for such observations remains to be explored.

Thus, we report that selection for late evening eclosion in fruit flies *D. melanogaster* is associated with the coevolution of several life-history traits in the *late* populations, while no difference was observed between the *early*, the *early-control* and the *late-control* populations*.* Such correlations between chronotypes/circadian clocks and life-history traits have been reported earlier. Notably, Yadav and Sharma [59, 60] demonstrated that selection for faster pre-adult development leads to the coevolution of shorter clock period, and that the faster developing populations evolve reduced dry-weight, body size, fecundity, starvation and desiccation resistance, and longevity. Similarly, in a separate study on the melon fly *Bactrocera cucurbitae*, selection for egg-to-adult duration resulted in the coevolution of divergent phase of activity/rest and mating rhythms [40]. Most differences observed in our study, however, correspond to the *late* populations relative to the *control* populations, while very little difference was observed between the *early* and the *control* populations. Also, most of the life-history traits assayed in the *late* populations differed by a small magnitude varying from 2-10 % as compared to the *early* and the *control* populations. This is not surprising considering the larger time difference between the selection window of the *late* populations from the eclosion peak of the *control* populations, and proximity of the selection window of the *early* populations from the eclosion peak of the *control* populations (see materials and methods). Since evening eclosion is not predominantly seen in the *control* populations, the *late* populations would experience a much stronger selection pressure as compared to the *early* populations which in turn might drive faster coevolution of life-history traits.

In summary, selection for late evening eclosion leads to lengthening of pre-adult duration without any increase in body-weight at eclosion, increased fecundity associated with greater post-fecundity assay dry-weight loss and reduced virgin female longevity. The observed life-history traits in the *late* populations being evolved responses to selection is further supported by our observation on the *early-control* and the *late-control* populations. That life-history traits of the *early-control* and the *late-control* populations did not differ significantly from each other but were different from that of the *early* and the *late* populations (in most cases) suggests that the observed life-history trait differences between the *early* and the *late* populations are evolutionary response to the imposed selection and are not merely environmentally driven. Furthermore, although the pre-adult and adult life-history traits studied here are known to be highly correlated, enhanced fecundity in the *late* populations does not seem to be a consequence of higher biomass attained by lengthening of egg-to-adult duration. Thus the differences in adult traits do not seem to be associated with pre-adult trait differences and appear to be driven by independent mechanisms that might have evolved as a consequence of selection.

## Conclusions

Thus, in contrast to studies which demonstrated the effect of direct manipulation of circadian clock on fitness aspects (see introduction), we report coevolution of life-history traits in independently evolved replicate populations of *D. melanogaster* exhibiting *early* and *late* eclosion chronotypes, suggesting that the genetic architecture underlying eclosion at specific times of the day (eclosion chronotypes) is genetically correlated with several life-history traits, and these correlations appear to encompass both circadian clock-dependent and clock-independent mechanisms. Thus the extent of circadian clocks’ influence in the observed trait differences, and the underlying genetic architecture remains to be explored.

## Methods

### Experimental populations and assay conditions

Additional file 1: Figure S2 presents a schematic of the selection protocol employed to generate the *early* and the *late* populations from the *control* populations. Populations selected for early morning and late evening eclosion comprised four replicates each of the *early*_i_ and the *late*_j_ (*i* = *j* = 1-4) initiated from four replicates of the *control*_k_ (*k* = 1-4) whose ancestry details are provided elsewhere [36]. Briefly, the *early* and the *late* populations with a given subscript were derived from the *control* population with the same subscript, and therefore share a common ancestry. For example, the *early*_1_ and the *late*_1_ populations were initiated from the *control*_1_ population, and similarly for the other three replicates. Since our study aims at exploring evolutionary trajectories of traits in a population, the unit of biological replication is a population and thus, the four populations of each selection type are biological replicates in all our experiments. All 12 populations (four replicates each for *early*, *control* and *late*) were maintained on a 21 day discrete generation cycle, and flies were housed in plexi-glass cages of dimension 25 × 20 × 15 cm^3^ with ~1200 individuals per cage (sex ratio ~1:1), and were provided with *ad libitum* banana-jaggery (BJ) medium. The parental populations were provided with food supplemented with yeast paste (to boost their fecundity) for three days prior to egg collection, and ~300 eggs were collected and dispensed into each culture vial (16 vials for *control,* 24 for *early* and 48 for *late* populations) containing ~6 ml BJ medium. From the initiation of eclosion, which is generally on day 9 (at 25 °C) post egg collection, flies that eclosed early in the morning between Zeitgeber Time (ZT) 21-01 (ZT00 and ZT12 represents time of lights-ON and lights-OFF respectively under LD12:12) for 3-4 consecutive days were collected to form the *early* populations, while flies that eclosed late in the evening between ZT09-13 formed the *late* populations. For the *control* populations, flies were collected once every 24 h for the same 3-4 days and thus, comprised individuals emerging throughout these 3-4 days without any selection imposed on timing of eclosion. The days of initiation and termination of fly collection within the respective selection windows was kept constant for all populations. In other words, if collection of flies for the *early* populations was started on day x and terminated on day y, collection of flies for the *control* and the *late* populations was also initiated and terminated on days ‘x’ and ‘y’ respectively so as to ensure that the populations are selected only for eclosion at different gate/time of the day and to avoid any unintended selection for faster and slower pre-adult development. The implementation of selection protocol and regular maintenance of populations was performed under LD12:12 with ~0.4 Wm^-2^ light intensity during the light phase, 25 ± 0.5 °C temperature, and 75 ± 5 % relative humidity.

In addition to the four replicate populations each for the *early*, the *control* and the *late*, we used four replicates each for the two other populations (*early-control* and *late-control*; see Introduction). From the *control* populations, flies emerging in the morning window (ZT21-01) were collected to form the *early-control* populations and similarly, flies emerging in the evening window (ZT09-13) formed the *late-control* populations. This procedure was implemented on all four replicates of the *control* populations for only one generation prior to the assays, and therefore, unlike the *early* and the *late* populations, the *early-control* and the *late-control* populations were not subjected to any long-term selection protocol.

To minimize the effects of non-genetic inheritance (reviewed in [61]) due to different selection regimes, all populations were subjected to one generation of standardization with the maintenance protocol which is identical to that used for the *control* populations. This was achieved by relaxing selection on timing of eclosion by collecting all flies that eclosed throughout the first 4 days similar to that for the *control* populations while the population size was maintained at ~1200 flies per replicate population. Since the primary purpose of using the *early-control* and the *late-control* populations was to asses if the observed differences between the *early* and the *late* populations are evolved responses to selection as not merely environmental in origin, these populations were also subjected to standardization by deriving them from the *control* populations followed by relaxation of selection for one generation as described above. All assays described in the present study were performed on the progeny of the standardized populations at the 242^nd^ generation (~14 years) either in LD12:12 or DD, or both, with light intensity, temperature and humidity same as that for the maintenance of populations. Fly handling and experiments in the dark were performed under dim red light (*λ* > 650 nm).

#### Egg-to-puparium duration assay

Egg-to-puparium duration for all the populations was assayed under two light regimes ─ LD12:12 and DD. After having provided yeast paste supplemented media for three days, all populations were provided with media plates for 1 h as substrate for oviposition. These plates were then replaced by fresh food medium plates for the next 1 h. Eggs laid on these plates were collected and 30 eggs were dispensed into each vial. A total of 10 such vials were used per replicate population per light regime making a total of 300 eggs per population per light regime. These vials were transferred to respective light regimes and monitored for the first pupariation event. After the first puparium was observed, vials were checked every two hours to count the number of puparia formed thereafter, and the assay was terminated when no pupariation event was seen for 24 consecutive hours. It was observed that a small proportion of larvae took relatively longer to pupariate, thus rendering the egg-to-puparium duration distribution right skewed (Fig. 1a, b; Additional file 1: Figure S3). Mean egg-to-puparium duration cannot be used as a reliable measure for such distributions [62], and therefore, we used median egg-to-puparium duration (calculated as the time from egg collection for 50 % of total pupariation events in a vial) for the same. The median egg-to-puparium duration was estimated for every replicate vial and then averaged across vials to obtain median egg-to-puparium duration for a given replicate population.

#### Egg-to-adult duration assay

Egg collection protocol and environmental conditions for the egg-to-adult duration assay were identical to egg-to-puparium duration assay. After egg collection and transfer to LD12:12 or DD, eclosion of the first adult fly was monitored following which vials were subjected to two hourly checks to count the number of flies that eclosed thereafter. The assay was terminated when no eclosion event was observed for 24 h. To facilitate comparisons between egg-to-puparium and egg-to-adult duration, we used median durations as a measure for analysis. The procedure to estimate median egg-to-adult duration was same as that described for median egg-to-puparium duration.

#### Estimation of egg-to-puparium and egg-to-adult survivorship

Egg-collection protocol and environmental conditions for the survivorship assays were same as that for the egg-to-puparium and egg-to-adult duration assay. Proportion of 30 eggs (total number of eggs dispensed per vial for the assay) that successfully pupariated was used as a measure for egg-to-puparium survivorship, while proportion of adults that successfully eclosed was used to estimate egg-to-adult survivorship. Individuals that were stuck in the pupal case and died within the pupa were considered to not have eclosed successfully. Percentage survivorship was calculated for every replicate vial and then averaged across vials to obtain average survivorship per replicate population.

#### Dry-weight at pupariation

The protocol for egg collection and subsequent environmental conditions for development under LD12:12 and DD was the same as that described for egg-to-puparium duration assay. From the initiation of the first pupariation event, freshly formed puparia (P1 stage) were collected every 2 h and frozen at -20 °C. These puparia were later sorted into 10 replicate groups with 5 puparia in each group; dried at 70 °C for 36 h after which their dry weight was assayed. Dry-weight of each group was measured at least thrice to account for instrument error and then normalized by the number of puparia (*n* = 5). The dry-weight measurements from 10 such groups were then averaged to obtain mean puparium dry-weight per replicate population.

#### Dry-weight at eclosion

The protocol for assaying dry-weight at eclosion was the same as that for dry-weight at pupariation assay except that instead of puparia freshly eclosed (within 2 h of eclosion) adult flies in LD12:12 or DD were used.

#### Fecundity assay

The populations used in the present study are maintained on a 21 day discrete generation cycle where eggs for the next generation are collected on day 21 post egg collection (average adult age of 11 days). Since only eggs laid around this day would determine an individual’s contribution to the gene pool for the next generation and consequently to its fitness, we estimated fecundity only under LD12:12 around day 11 (post-eclosion) in the progeny of standardized populations, which were collected in plexi-glass cages and maintained under LD12:12 in mixed-sex groups similar to that used for regular maintenance of populations. On day 8 (average adult age), flies from plexi-glass cages were collected, separated using mild carbon-di-oxide anesthesia and transferred into vials containing ~4 ml BJ media for conditioning at a density of 10 flies/vial (5 of each sex). In parallel, additional sets of conditioning vials were set aside from which flies for pre-fecundity dry-weight assay were to be collected later (described in the following section). On day 10, flies from the conditioning vials were sorted into single male–female pairs and transferred into 20 vials/population containing 1 ml BJ medium. After 24 h (day 11), flies were transferred to fresh set of vials and the same was repeated on day 12. Average number of eggs laid per female across days 10-12 was used as a measure of mean fecundity/female around day 11. Only vials from which data could be collected for all three days were used and those in which either male or female died within the three days were not used for data analysis.

#### Estimation of pre- and post-fecundity assay dry-weights

To assess pre-fecundity assay dry-weight of females, 20 females (for every replicate population) from separate sets of conditioning vials (which were not used for fecundity assay) as described in the preceding section were frozen at -20 °C at the beginning of day 10. Additionally, at the end of the fecundity assay (end of day 12), females used for the assay were collected and frozen. All flies were then dried at 70 °C for 36 h, sorted into groups of 5 individuals each and weighed at least thrice to estimate dry-weight/female. Dry-weight measurements were then averaged across groups to calculate mean pre- and post-fecundity assay dry-weight/female/replicate population. Further, dry-weight loss during fecundity assay was estimated by calculating the difference in pre- and post-fecundity assay dry-weight and was used to normalize the fecundity/female values to calculate fecundity per unit dry-weight lost as an estimate for biomass to egg conversion ratio. However, this is under the assumption that the biomass lost is entirely converted to eggs laid which may not necessarily be the case but nevertheless can be used as a proxy for assessment of for biomass-to-egg conversion ratio.

#### Longevity assay

Longevity of flies was assayed only in LD12:12 with environmental conditions same as described previously. Freshly eclosed virgin males and females were collected from the progeny of standardized populations every 6 h over three consecutive days. On the fourth day, all flies of a given sex and population were mixed and randomly distributed in groups of 10 flies/vial/sex into 10 replicate vials containing ~4 ml BJ media. Therefore, every replicate population comprised 20 vials in total with 10 vials for each sex and each vial housing 10 flies (average age of 2 days). Thereafter, flies were transferred to fresh BJ media every 3rd day and longevity was estimated by counting the number of dead flies in each vial every 24 h. The assay was continued until all flies died. While care was taken to ensure no flies escaped during transfers to fresh media vials, a few of them either escaped or were crushed between the cotton plug and the vial, and hence were not considered for calculating percentage survivorship for that vial. Similar to egg-to-puparium and egg-to-adult duration, longevity distribution was also right-skewed and therefore, we used median longevity (time taken for the death of 50 % of individuals in a given vial) as the measure of longevity.

### Statistical analyses

All measures of pre-adult duration, pre-adult survivorship, fecundity, dry-weight and longevity were estimated for each replicate vial and then averaged to obtain mean values for replicate populations. These replicate means served as data for statistical analyses by a randomized block design mixed model analysis of variance (ANOVA) with ‘population’, ‘light regime’, ‘stage (at which fecundity was assayed)’ or ‘sex’ (whichever was appropriate) as fixed factors and ‘replicate population’ as random factor. All percentage and ratio values were arcsine square root and log transformed respectively before subjecting them to ANOVA. Post hoc multiple comparisons were performed at a significance level (*α*) of 0.05 by method of Tukey’s HSD. All statistical analyses were implemented on STATISTICA for Windows, Release 5.0B (Statsoft 1995).

## Availability of data and materials

The dataset(s) supporting the conclusions of this article is(are) included within the article and as supplementary online material.

## Additional file

Additional file 1:Figure S1. Schematic of eclosion profile of *D. melanogaster* under laboratory LD12:12 (12 h of light and dark each) cycles at 25 °C. The shaded area represents night and the unshaded area represents day. Zeitgeber Time (ZT) depicts the time of day with ZT00 indicating lights-ON and ZT12 representing lights-OFF. **Figure S2:** Schematic of laboratory selection protocol employed for the *early* and the *late* populations. Zeitgeber Time (ZT) 21-00 represents the early window during which flies for the *early* populations are collected and ZT09-13 represents the late window during which flies for the *late* populations are collected. **Figure S3:** Proportion of individuals pupariated as a function of time from egg collection for the *early* (panel 1), the *early-control* (panel 2), the *control* (panel 3), the *late-control* (panel 4) and the *late* (panel 5) populations in (a) LD12:12 and (b) DD. R1-R4 represents the four replicates of the respective populations used for the study. The black and white horizontal bars at the bottom represent night and day respectively. **Figure S4:** Proportion of individuals eclosed as a function of time from egg collection for the *early* (panel 1), the *early-control* (panel 2), the *control* (panel 3), the *late-control* (panel 4) and the *late* (panel 5) populations in (a) LD12:12 and (b) DD. R1-R4 represents the four replicates of the respective populations used for the study. The black and white horizontal bars at the bottom represent night and day respectively. **Table S1.** Median egg-to-puparium and egg-to-adult duration presented as mean (± SD) in hours for all populations in LD12:12 and DD light regimes. **Table S2:** Percentage egg-to-puparium survivorship and egg-to-adult survivorship presented as mean (± SD) for all populations in LD12:12 and DD light regimes. **Table S3:** Average dry-weight at pupariation and at eclosion presented as mean (± SD) in μg for all populations in LD12:12 and DD light regimes. **Table S4:** Average eggs laid/female on day 11 post-eclosion, dry-weight in μg at pre- and post-fecundity assay stages, and median longevity of all populations in LD12:12. All values are presented as mean (± SD). (PDF 14678 kb)
