# Supplementary material for: Life-history traits of Drosophila melanogaster populations exhibiting early and late eclosion chronotypes
Source: BMC Evol Biol. 2016 Feb 27;16:46. doi: 10.1186/s12862-016-0622-3 (PMC4769836; doi:10.1186/s12862-016-0622-3)
Supplement: Additional file 1:Figure S1. — Schematic of eclosion profile of D. melanogaster under laboratory LD12:12 (12 h of light and dark each) cycles at 25 °C. The shaded area represents night and the unshaded area represents day. Zeitgeber Time (ZT) depicts the time of day with ZT00 indicating lights-ON and ZT12 representing lights-OFF. Figure S2: Schematic of laboratory selection protocol employed for the early and the late populations. Zeitgeber Time (ZT) 21-00 represents the early window during which flies for the early populations are collected and ZT09-13 represents the late window during which flies for the late populations are collected. Figure S3: Proportion of individuals pupariated as a function of time from egg collection for the early (panel 1), the early-control (panel 2), the control (panel 3), the late-control (panel 4) and the late (panel 5) populations in (a) LD12:12 and (b) DD. R1-R4 represents the four replicates of the respective populations used for the study. The black and white horizontal bars at the bottom represent night and day respectively. Figure S4: Proportion of individuals eclosed as a function of time from egg collection for the early (panel 1), the early-control (panel 2), the control (panel 3), the late-control (panel 4) and the late (panel 5) populations in (a) LD12:12 and (b) DD. R1-R4 represents the four replicates of the respective populations used for the study. The black and white horizontal bars at the bottom represent night and day respectively. Table S1. Median egg-to-puparium and egg-to-adult duration presented as mean (± SD) in hours for all populations in LD12:12 and DD light regimes. Table S2: Percentage egg-to-puparium survivorship and egg-to-adult survivorship presented as mean (± SD) for all populations in LD12:12 and DD light regimes. Table S3: Average dry-weight at pupariation and at eclosion presented as mean (± SD) in μg for all populations in LD12:12 and DD light regimes. Table S4: Average eggs laid/female on day 11 post-eclosion, dry-weight in μg a [file 12862_2016_622_MOESM1_ESM.pdf]

SUPPLEMENTARY ONLINE MATERIAL

Life-history traits of *Drosophila melanogaster* populations exhibiting *early* and *late* eclosion chronotypes

KL Nikhil, Karatgi Ratna and Vijay Kumar Sharma*

Chronobiology Laboratory, Evolutionary and Organismal Biology Unit, Jawaharlal Nehru Centre for Advanced Scientific Research, Jakkur, PO Box. 6436, Bangalore-560064, Karnataka, India.

Running Title: *Chronotypes and life-history traits.*

***Correspondence to:** Vijay Kumar Sharma

E-mail: vsharma@jncasr.ac.in/ vksharmas@gmail.com; **Phone:** +91-080-22082843 (office)/ 2844/45 (lab); **Fax:** +91-080-22081766; **Url:** http://www.jncasr.ac.in/vsharma.

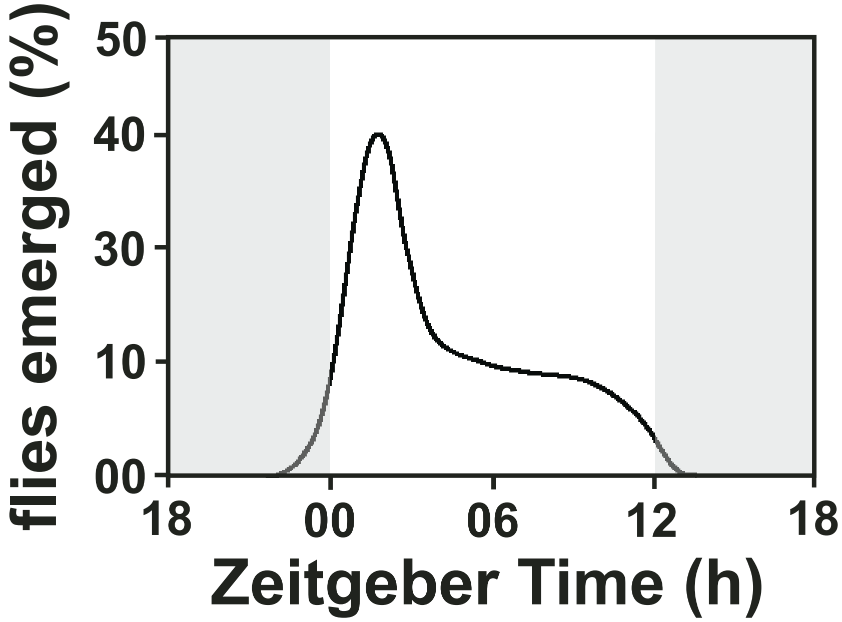

Supplementary Figure S1: Schematic of eclosion profile of *D. melanogaster* under laboratory LD12:12 (12 h of light and dark each) cycles at 25 ^{o}C. The shaded area represents night and the unshaded area represents day. Zeitgeber Time (ZT) depicts the time of day with ZT00 indicating lights-ON and ZT12 representing lights-OFF.

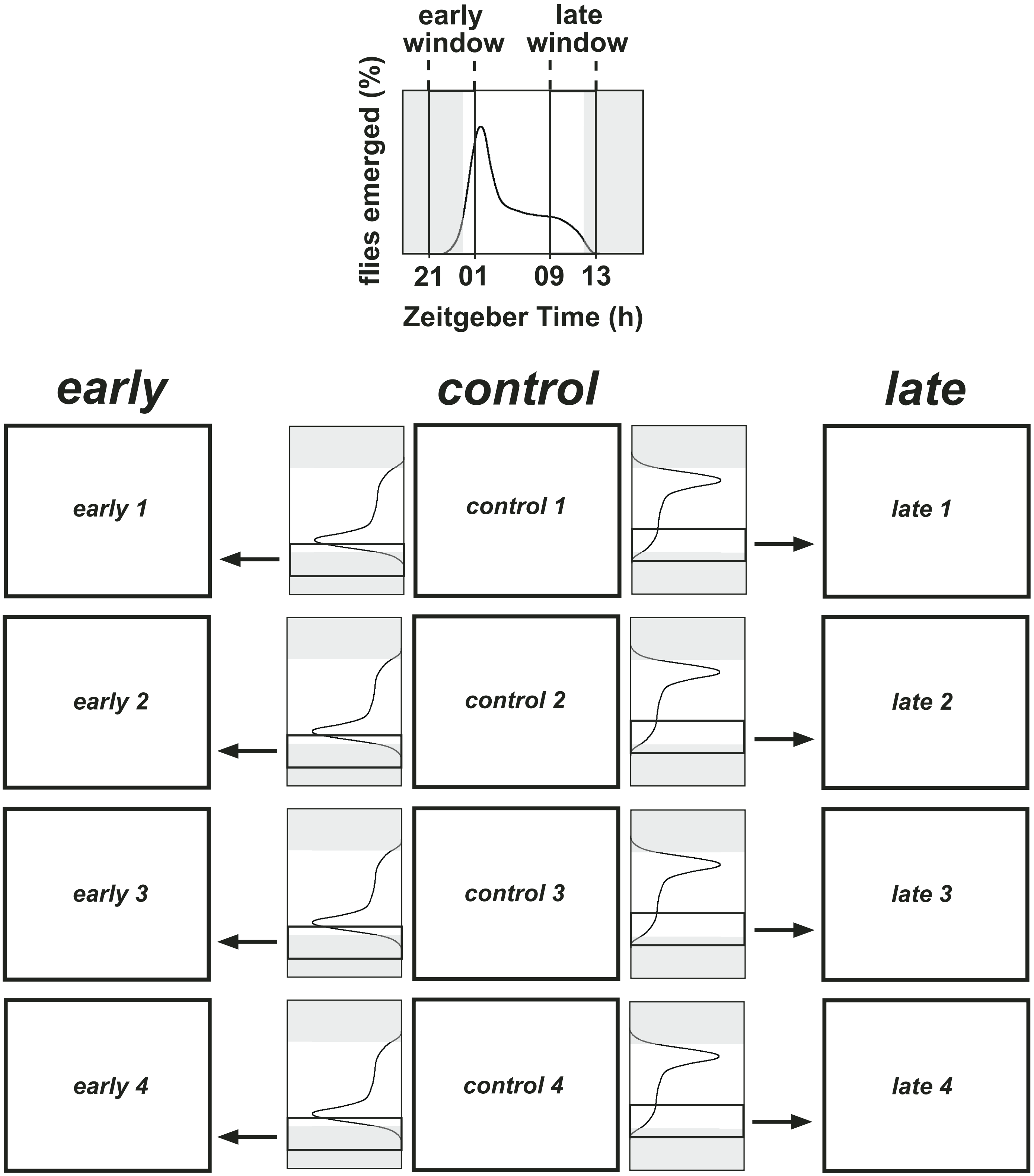

Supplementary Figure S2: Schematic of laboratory selection protocol employed for the *early* and the *late* populations. Zeitgeber Time (ZT) 21-00 represents the early window during which flies for the *early* populations are collected and ZT09-13 represents the late window during which flies for the *late* populations are collected.

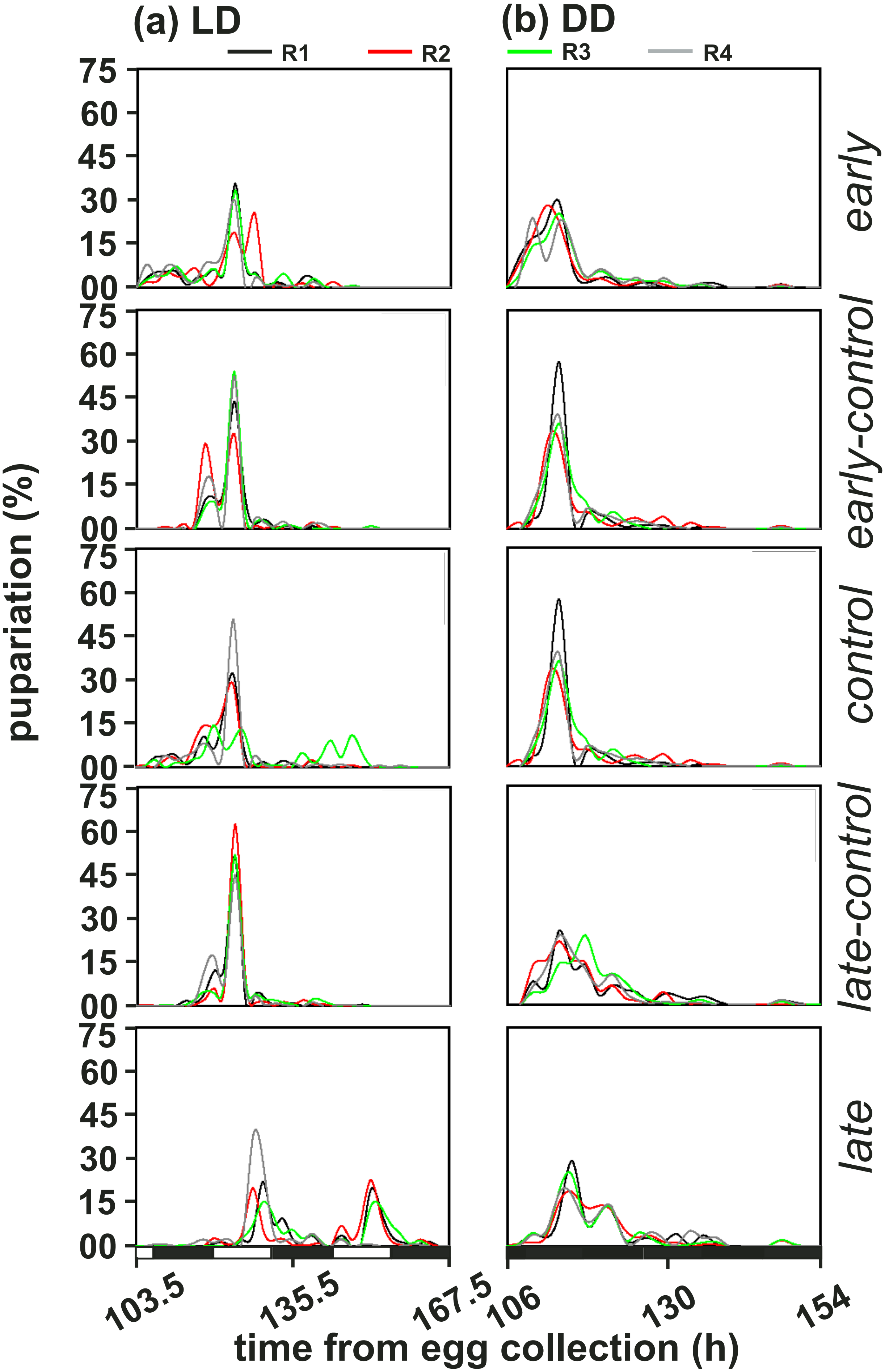

Supplementary Figure S3: Proportion of individuals pupariated as a function of time from egg collection for the *early* (panel 1), the *early-control* (panel 2), the *control* (panel 3), the *late-control* (panel 4) and the *late* (panel 5) populations in (a) LD12:12 and (b) DD. R1-R4 represents the four replicates of the respective populations used for the study. The black and white horizontal bars at the bottom represent night and day respectively.

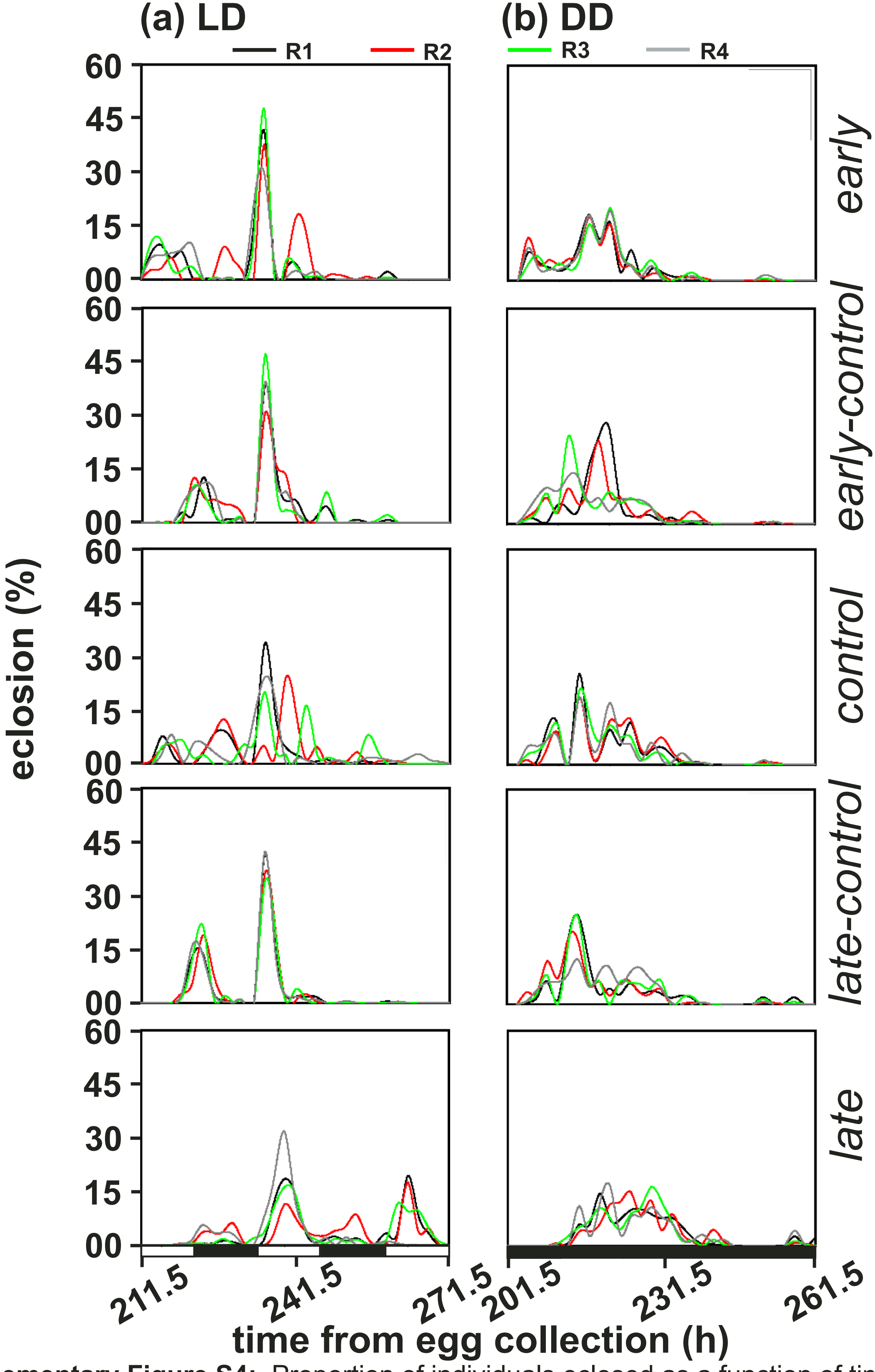

Supplementary Figure S4: Proportion of individuals eclosed as a function of time from egg collection for the *early* (panel 1), the *early-control* (panel 2), the *control* (panel 3), the *late-control* (panel 4) and the *late* (panel 5) populations in (a) LD12:12 and (b) DD. R1-R4 represents the four replicates of the respective populations used for the study. The black and white horizontal bars at the bottom represent night and day respectively.

Supplementary Table S1. Median egg-to-puparium and egg-to-adult duration presented as mean (± SD) in hours for all populations in LD12:12 and DD light regimes.

		**median egg-to-puparium duration**		**median egg-to-adult duration**	
**population**	**replicate**	**LD12:12**	**DD**	**LD12:12**	**DD**
early (E)	*1*	122.75 (1.48)	112.80 (1.39)	234.50 (1.06)	218.66 (1.73)
	2	123.50 (1.63)	113.00 (1.51)	235.50 (0.00)	217.50 (1.41)
	3	123.50 (1.15)	114.20 (0.66)	235.00 (0.92)	220.44 (1.33)
	4	120.00 (1.41)	114.25 (1.67)	233.78 (0.75)	219.00 (2.39)
early-control (EC)	*1*	123.50 (0.00)	116.57 (2.76)	235.90 (0.89)	220.25 (1.66)
	2	121.90 (1.67)	114.50 (2.33)	235.90 (0.89)	219.55 (1.33)
	3	123.16 (0.81)	114.28 (0.75)	235.50 (0.00)	216.28 (1.38)
	4	123.50 (0.00)	114.00 (0.00)	235.50 (0.00)	215.75 (2.49)
control (C)	*1*	121.72 (2.10)	115.00 (1.06)	235.21 (0.75)	218.50 (2.97)
	2	121.16 (1.50)	114.66 (1.63)	238.17 (1.63)	222.57 (2.76)
	3	121.25 (2.25)	113.75 (1.67)	237.75 (2.49)	217.00 (2.61)
	4	123.25 (0.70)	114.50 (1.41)	234.64 (1.06)	218.66 (2.64)
late-control (LC)	*1*	122.83 (1.48)	116.89 (3.17)	234.70 (2.53)	217.71 (2.13)
	2	123.78 (0.75)	114.40 (1.57)	234.25 (3.53)	215.11 (1.45)
	3	123.05 (1.33)	118.00 (1.06)	235.50 (0.00)	218.33 (3.20)
	4	121.90 (2.27)	116.00 (1.41)	234.30 (3.79)	220.00 (3.38)
late (L)	*1*	129.90 (1.57)	119.20 (3.55)	240.10 (0.96)	226.66 (3.60)
	2	127.72 (0.66)	118.22 (1.20)	238.30 (2.52)	226.75 (2.12)
	3	130.10 (0.96)	117.14 (1.57)	239.70 (1.13)	226.22 (3.23)
	4	128.75 (1.03)	118.00 (1.85)	238.50 (1.07)	226.00 (2.82)

Supplementary Table S2: Percentage egg-to-puparium survivorship and egg-to-adult survivorship presented as mean (± SD) for all populations in LD12:12 and DD light regimes.

population	replicate	egg-to-puparium survivorship		egg-to-adult survivorship	
		LD12:12	DD	LD12:12	DD
early (E)	*1*	84.16 (08.86)	81.00 (13.15)	82.50 (09.21)	80.00 (12.13)
	2	89.52 (07.31)	78.33 (09.59)	85.55 (09.81)	83.75 (06.77)
	3	88.33 (09.92)	87.40 (05.95)	87.08 (10.14)	85.55 (09.57)
	4	85.41 (17.45)	87.50 (07.50)	86.19 (09.89)	81.66 (09.08)
early-control (EC)	*1*	82.22 (09.00)	76.19 (18.70)	89.33 (07.95)	85.41 (09.74)
	2	87.33 (08.62)	86.66 (09.42)	86.66 (10.54)	84.07 (14.69)
	3	80.00 (08.94)	89.04 (07.62)	85.55 (09.35)	80.00 (07.93)
	4	84.66 (11.92)	80.83 (10.80)	84.00 (10.64)	80.00 (10.54)
control (C)	*1*	87.03 (03.88)	92.91 (09.16)	85.23 (09.97)	89.58 (14.52)
	2	87.22 (18.57)	90.95 (09.56)	84.44 (10.88)	87.14 (09.11)
	3	83.75 (06.77)	82.85 (09.70)	84.58 (12.46)	85.00 (11.12)
	4	85.41 (09.58)	85.41 (10.97)	83.80 (08.62)	85.55 (09.42)
late-control (LC)	*1*	84.07 (06.82)	77.77 (15.89)	84.66 (06.70)	79.52 (06.78)
	2	86.66 (08.35)	88.00 (08.91)	86.25 (04.86)	86.66 (09.72)
	3	85.92 (13.09)	82.50 (14.45)	84.44 (07.81)	78.33 (12.78)
	4	83.66 (15.10)	85.18 (14.04)	82.66 (18.13)	85.55 (14.90)
late (L)	*1*	87.66 (06.85)	82.66 (10.16)	84.66 (14.33)	80.37 (09.19)
	2	84.44 (11.66)	87.40 (08.46)	87.00 (15.59)	82.96 (10.19)
	3	88.00 (09.83)	91.90 (07.66)	89.66 (10.82)	89.63 (08.06)
	4	87.62 (06.58)	92.50 (10.94)	84.58 (10.94)	78.09 (12.74)

Supplementary Table S3: Average dry-weight at pupariation and at eclosion presented as mean (± SD) in μg for all populations in LD12:12 and DD light regimes.

population	replicate	dry-weight at pupariation		dry-weight at eclosion	
		LD12:12	DD	LD12:12	DD
early (E)	*1*	564.26 (11.56)	538.65 (13.21)	360.85 (08.49)	345.33 (10.65)
	2	588.96 (16.25)	536.35 (15.41)	364.41 (15.16)	347.18 (12.39)
	3	585.92 (16.16)	519.33 (12.03)	361.98 (10.55)	338.26 (11.04)
	4	565.45 (15.42)	539.74 (10.50)	350.33 (11.96)	339.75 (09.53)
early-control (EC)	*1*	564.13 (19.09)	535.71 (12.29)	364.46 (15.19)	344.14 (09.18)
	2	573.60 (18.05)	542.43 (19.62)	352.95 (12.64)	345.18 (13.30)
	3	576.16 (19.68)	536.47 (15.94)	353.26 (11.47)	345.98 (10.92)
	4	568.21 (20.39)	531.28 (19.13)	362.51 (07.97)	356.08 (12.99)
control (C)	*1*	571.64 (18.55)	539.77 (14.53)	358.01 (12.40)	348.40 (16.00)
	2	565.35 (16.66)	534.71 (08.61)	358.60 (07.96)	351.93 (10.27)
	3	571.38 (18.82)	536.93 (18.54)	360.31 (10.57)	339.48 (09.99)
	4	580.10 (15.51)	521.89 (19.46)	369.43 (08.65)	344.95 (10.43)
late-control (LC)	*1*	565.93 (20.90)	546.58 (15.21)	362.00 (11.88)	348.30 (10.72)
	2	581.53 (17.81)	542.20 (18.74)	359.46 (10.95)	343.68 (14.34)
	3	572.73 (11.76)	540.00 (17.01)	366.03 (11.71)	348.60 (12.41)
	4	580.50 (07.98)	547.50 (09.62)	363.06(12.64)	351.93 (06.10)
late (L)	*1*	580.50 (14.46)	584.40 (11.10)	371.86 (16.61)	347.80 (09.09)
	2	583.14 (15.30)	549.00 (10.36)	367.44 (11.08)	350.02 (08.96)
	3	575.64 (19.31)	539.55 (09.04)	366.86 (11.50)	341.20 (06.24)
	4	581.12 (16.41)	542.37 (14.03)	373.41 (13.67)	353.23 (06.31)

Supplementary Table S4: Average eggs laid /female on day 11 post-eclosion, dry-weight in µg at pre- and post-fecundity assay stages, and median longevity of all populations in LD12:12. All values are presented as mean (± SD).

population	replicate	eggs laid/female	dry-weight		median longevity	
			pre-fecundity	post-fecundity	female	males
early (E)	*1*	7.05 (2.47)	237.50 (7.03)	180.00 (2.83)	41.00 (3.09)	47.16 (3.76)
	2	6.74 (1.97)	227.66 (5.47)	182.00 (2.29)	41.50 (6.41)	46.00 (5.23)
	3	7.59 (2.42)	229.83 (2.03)	179.16 (4.45)	42.10 (4.70)	47.83 (3.43)
	4	7.90 (2.21)	232.83 (3.18)	190.00 (6.30)	41.00 (5.52)	49.00 (3.76)
early-control (EC)	*1*	8.33 (3.06)	225.00 (3.45)	185.16 (6.18)	45.50 (1.73)	45.00 (3.77)
	2	7.23 (2.71)	234.83 (2.79)	187.00 (4.86)	40.83 (3.81)	46.40 (6.06)
	3	7.79 (2.78)	237.16 (4.27)	183.20 (1.48)	42.71 (3.59)	46.42 (5.41)
	4	7.62 (1.71)	237.50 (6.37)	181.83 (1.05)	41.66 (7.60)	47.71 (4.71)
control (C)	*1*	7.17 (1.97)	232.16 (4.00)	190.33 (7.48)	43.16 (7.13)	47.85 (5.04)
	2	6.96 (2.79)	232.50 (5.58)	188.83 (4.65)	44.37 (5.95)	48.88 (5.51)
	3	6.56 (2.11)	228.66 (1.38)	189.66 (9.74)	43.80 (1.92)	47.50 (3.11)
	4	7.37 (1.80)	226.50 (8.50)	184.83 (4.70)	45.66 (3.20)	47.20 (3.39)
late-control (LC)	*1*	7.85 (2.32)	238.50 (1.02)	184.83 (1.32)	44.50 (3.73)	44.25 (3.91)
	2	6.41 (3.64)	237.83 (6.76)	185.50 (3.44)	45.37 (5.20)	45.57 (3.55)
	3	6.85 (2.46)	228.66 (1.38)	177.00 (1.50)	41.83 (6.08)	46.57 (4.27)
	4	9.62 (3.72)	226.50 (8.50)	181.33 (3.98)	44.83 (8.63)	44.50 (3.93)
late (L)	*1*	10.7 (2.84)	233.16 (5.65)	163.66 (1.09)	42.83 (6.17)	42.42 (5.02)
	2	10.3 (3.47)	240.50 (2.82)	167.33 (7.00)	41.14 (6.38)	42.00 (2.89)
	3	11.0 (1.90)	241.16 (2.67)	168.16 (4.79)	40.60 (6.73)	40.60 (8.82)
	4	11.0 (2.24)	238.00 (3.72)	169.50 (1.60)	43.00 (3.28)	42.57 (3.25)
